# The PAHO/WHO Regional Network of Interprofessional Health Education

**DOI:** 10.1590/1518-8345.0000.2866

**Published:** 2017-10-19

**Authors:** Sabrina de Souza Elias Mikael, Silvia Helena De Bortoli Cassiani, Fernando Antonio Menezes da Silva

**Affiliations:** 1 Sabrina de Souza Elias Mikael is MSc, International Consultant, Unit of Human Resources for Health (HSS/HR), Department of Health Systems and Services (HSS), Pan American Health Organization/World Health Organization (PAHO/WHO), Washington, DC, United States of America. Email: desouzas@paho.org; 2 Silvia Helena De Bortoli Cassiani is PhD, Regional Advisor on Nursing and Allied Health Personnel, Pan American Health Organization/World Health Organization (PAHO/WHO), Washington, DC, United States of America. Email: cassianis@paho.org; 3 Fernando Antonio Menezes da Silva is PhD, Unit Chief, Unit of Human Resources for Health (HSS/HR), Department of Health Systems and Services (HSS), Pan American Health Organization/World Health Organization (PAHO/WHO), Washington, DC, United States of America. Email: menezesf@paho.org

**1:**
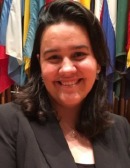

**2:**
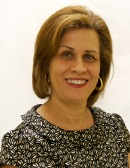

**3:**
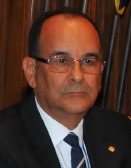

In 2014, at the 53^rd^ Directing Council of the Pan American Health Organization/World Health Organization (PAHO/WHO), the countries of the Region of the Americas reaffirmed their commitment to the Strategy of Universal Access to Health and Universal Health Coverage (Universal Health)^1^. Improvements in primary care resolution capacity, and the distribution, training, and qualification of human resources for health, are important factors in achieving Universal Health in the Region^2^.

There are several obstacles to access universal, comprehensive, good-quality health services in the Region of the Americas^1^. Despite advances in economic and social development and the consequent strengthening of health systems in the Region, collaboration across health, education, and labor sectors needs to be better aligned to promote education that will prepare professionals to meet the health needs of the population and the countries^3^ ^-^ ^4^.

Interprofessional collaboration is a promising strategy for mitigating the workforce crisis and improving health care that, if carried out by interprofessional health teams, enables optimization of skills and holistic, high-quality, people-centered care^5^ ^-^ ^6^.

Effective collaboration among health team members requires health professional education based on the interprofessional education (IPE) approach. As defined by the WHO, IPE “occurs when students from two or more professions learn about, from and with each other to enable effective collaboration and improve health outcomes”^5^.

Evidence indicates that IPE 1) promotes the development of attitudes, knowledge, skills, and behaviors conducive to collaborative practice and 2) improves teamwork by developing respect for and recognition of individuals’ skills^7^. This type of training enables health professionals to use the full capacity of their training^3^ ^-^ ^4^ ^,^ ^8^. If used throughout professional training rather than as isolated components of the educational curriculum, IPE can strengthen health sector human resources capacity, improve outcomes, and thus strengthen health systems^5^ ^-^ ^9^. Therefore, WHO recommends that educational establishments adapt their institutional structures and teaching modalities to promote both IPE and collaborative practice^3^.

In 2016, PAHO/WHO held a meeting in Bogota, Colombia (“Interprofessional Education in Healthcare: Improving Human Resources Capacity to Achieve Universal Health”), to support countries in the Region in the implementation or strengthening of IPE. Meeting participants included representatives from ministries of health and education, academic institutions, school associations, and professional associations in 12 countries: Argentina, Brazil, Chile, Colombia, Costa Rica, Guatemala, Jamaica, Mexico, Panama, Peru, Trinidad and Tobago, and Uruguay.

With the support of IPE experts from Canada, Spain, the United Kingdom, and the United States, meeting participants discussed the rationale and foundation for IPE, as well as its theoretical, practical, and political frameworks; the individual and institutional attributes, resources, and commitments required for its implementation; faculty development for IPE; IPE curriculum development and implementation; the regulation of health professionals within the IPE context; and aspects related to interprofessional team management. Participants also presented and exchanged information about their experiences with IPE in various countries of the Region.

An important outcome of the meeting was the launch of the Regional Network of Interprofessional Health Education, coordinated by Argentina, Brazil, and Chile, with the participation of several other countries in the Region of the Americas. The regional network aims to 1) propose activities for developing IPE within the context of Universal Health; 2) provide a forum for the exchange and dissemination of IPE information, including experiences, knowledge, scientific evidence, as well as methods and resources for teaching and conducting research; 3) identify common IPE facilitators and barriers; 4) encourage the development of intersectoral and multicenter research; 5) monitor the status of IPE and related trends in health to facilitate priority setting related to IPE and its development; 6) support the articulation between interprofessional education and practice; and 7) monitor and support of countries activities. Additional meetings have already been held to discuss network activities and subsequent steps.

After the meeting in Bogotá, six countries-Argentina, Brazil, Chile, Colombia, Costa Rica, and Uruguay-presented action plans for implementing or developing activities to advance IPE through joint work between ministries of health, ministries of education, academic institutions, school associations, and professional associations. Proposed activities included analysis of the current status of IPE; conceptual alignment and dissemination; identification of interested partners for IPE projects/research; encouragement of IPE initiatives; faculty development for EIP; promotion of the IPE theme in continuing health education; IPE related knowledge production and dissemination; elaboration of interinstitutional agreements for IPE implementation; and the development of undergraduate and postgraduate courses that incorporate an interprofessional approach. The next IPE regional meeting to discuss the progress of the country’s action plans will be held in Brasilia in late 2017.

Much remains to be done to ensure that health professionals’ education is focused on the needs of the health systems and that trained professionals work effectively in health teams that are articulate and well-versed in people- and community-centered, integrated care^1^ ^,^ ^3^ ^-^ ^4^. The interest shown by countries of the Region in supporting and encouraging PAHO/WHO to continue the discussion on IPE could be another step toward building interprofessional health teams and could thus help improve the effectiveness of health systems and contribute to the achievement of Universal Access to Health and Universal Health Coverage.
